# Stress Reaction: Ozone and Co-pollutants Linked with Oxidation

**Published:** 2007-12

**Authors:** Bob Weinhold

Past studies have hinted that several air pollutants can cause oxidative stress in people, suggesting one mechanism behind diseases such as lung cancer, asthma, and increased cardiopulmonary illnesses and deaths. The evidence has gained more support with the discovery that chronic exposure to ambient ozone, nitrogen dioxide, or particulates is strongly linked with lipid peroxidation, an indicator of oxidative stress **[*EHP* 115:1732–1737; Chen et al.]**. Moreover, more damage was seen at higher pollutant concentrations.

Researchers evaluated two oxidative stress indicators in the blood of 120 University of California, Berkeley, students aged 18 to 22 years. Each student was a lifelong resident of either the Los Angeles or San Francisco area, where they had experienced variable seasonal exposures to pollutants including ozone.

To assess lipid peroxidation, the researchers measured 8-iso-prostaglandins-F_2α_ (8-iso-PGF), which has been found in several studies to be a useful indicator. To assess total antioxidant capacity, they measured ferric-reducing ability of plasma (FRAP), which has a more limited history as an indicator. Pollutant concentrations were estimated from data at monitoring stations near where the students lived.

The lifelong Los Angeles residents had received much higher average ozone exposure over the course of a lifetime (42.9 ppb versus 26.9 ppb for the San Francisco residents), and 8-iso-PGF was twice as high in these students (195.3 pg/mL versus 97.2 pg/mL). There was also a link between relatively higher ambient ozone concentrations during 2-week and 1-month periods and increased 8-iso-PGF. Neither sex, ethnicity, nor weight affected the results, but there was a wide range in 8-iso-PGF among individuals (17.4–940.7 pg/mL), perhaps due to genetic differences.

The researchers also found a significant link between 8-iso-PGF and increased concentration of nitrogen dioxide or particulates, independent of the effects of ozone.

The FRAP assay showed no significant difference in antioxidant capacity between the residents of the two cities. However, there was a threefold difference among all study subjects in antioxidant capacity, and males had about 23% more antioxidant capacity than females.

Although acknowledging that much more study of other populations and locations needs to be done with more precise measures of personal pollutant exposure, the researchers conclude that each of the three pollutants studied can significantly increase oxidative stress. They also report that 8-iso-PGF is an accurate indicator of oxidative stress, while noting that other indicators, such as cytogenetic damage, may also prove useful.

